# Health-related quality of life in nonvalvular atrial fibrillation patients with controlled or uncontrolled anticoagulation status

**DOI:** 10.1186/s12955-020-01563-1

**Published:** 2020-12-11

**Authors:** José Felipe Varona, José Miguel Seguí-Ripoll, Cristina Lozano-Duran, Luis Miguel Cuadrado-Gómez, Juan Bautista Montagud-Moncho, Antonio Ramos-Guerrero, José Carlos Mirete-Ferrer, Esther Donado, Javier García-Alegría, the REQUOL Study Group

**Affiliations:** 1 Hospital HM Montepríncipe, Boadilla del Monte, Madrid Spain; 2 Hospital San Juan de Alicante, San Juan de Alicante, Alicante Spain; 3 Department of Clinical Medicine, Miguel Hernández University, Elche, Alicante Spain; 4 Hospital Universitario Príncipe de Asturias, Alcalá de Henares, Madrid Spain; 5 Hospital Francesc de Borja, Gandía, Valencia Spain; 6 Hospital San Juan de Dios del Aljarafe, Bormujos, Seville Spain; 7 Hospital de Torrevieja, Torrevieja, Alicante Spain; 8 Boehringer-Ingelheim, Sant Cugat del Vallés, Barcelona Spain; 9 Hospital Costa del Sol, A-7, Km 187, 29603 Marbella, Malaga Spain

**Keywords:** Nonvalvular atrial fibrillation, Health-related quality of life, Vitamin K antagonists, Non-vitamin K antagonist oral anticoagulants, Anticoagulation control

## Abstract

**Background:**

There is a dearth of evidence regarding Health-Related Quality of Life (HRQoL) in nonvalvular atrial fibrillation (NVAF) patients undergoing oral anticoagulation therapy. Our objective was to describe HRQoL in NVAF patients on oral anticoagulation, focusing on uncontrolled patients on vitamin K antagonists (VKAs) versus controlled patients on VKAs or non-vitamin K antagonist oral anticoagulants (NOACs), in a real-world setting. Additionally, we assessed the clinical characteristics of patients with uncontrolled anticoagulation.

**Methods:**

An observational, multicentre, and cross-sectional study, enrolling 38 Spanish Hospitals' Internal Medicine Departments. HRQoL was assessed using the validated Spanish version of the Sawicki questionnaire. High self-perceived HRQoL was indicated by high scores in the general treatment satisfaction and self-efficacy dimensions, and by low scores in the strained social network, daily hassles and distress dimensions.

**Results:**

Five hundred and one patients were included for assessment. Mean scores ± SD were closer to a high perceived HRQoL in controlled than uncontrolled patients for the five dimensions of the questionnaire: 4.9 ± 1.0 versus 3.6 ± 1.3 for general treatment satisfaction; 4.3 ± 1.0 versus 3.6 ± 1.0 for self-efficacy, 3.1 ± 0.9 versus 3.9 ± 1.1 for strained social network, 2.1 ± 0.8 versus 3.0 ± 1.0 for daily hassles and 1.8 ± 0.9 versus 2.6 ± 1.2 for distress.

**Conclusions:**

HRQoL in patients with controlled anticoagulant status treated with NOACs or VKAs was better than in patients with uncontrolled anticoagulant status. This seems to indicate that anticoagulation control status influences perception of HRQoL, highlighting the importance of its evaluation when assessing HRQoL in NVAF patients.

## Background

Atrial fibrillation (AF) is the most common type of arrhythmia worldwide [1] and is associated with episodes of heart failure, cognitive decline, cardiovascular morbidity, an increased mortality risk and a decreased quality of life (QoL) [2–6]. The most serious common complication of AF are embolic events, including stroke [7], which usually result in severe disability and dependence [8]. The prevalence of AF in the general Spanish population over 40 years of age is high (4.4%), and it rises exponentially with age, reaching a prevalence of 17.7% in patients older than 80 years [9]. This higher prevalence in older adults has important implications for public health policy and health care costs, given the current demographic transition to an inverted age pyramid. The healthcare and economic burdens of AF are mainly driven by the high cost of hospital admissions, including those associated with stroke and bleeding complications [10, 11].

Oral anticoagulant therapy is effective for preventing stroke in patients with AF [12]. For many decades, the vitamin K antagonists (VKAs) have been the only oral anticoagulant drugs available for clinical use for the prevention of thromboembolic events. VKAs continue to be widely used in Spain, although there is a poor VKA anticoagulation control. Almost half of the patients are outside of the therapeutic range more than 50% of the time [13, 14], and women are at a higher risk of poor INR control [15], with the increased probability of thromboembolic events that uncontrolled INR implies.

These practical difficulties associated with VKAs led to the development of non-vitamin K antagonist oral anticoagulants (NOACs). NOACs maintain the benefits of anticoagulant therapy while overcoming some of the limitations of VKAs [16, 17]. They do not require strict monitoring, have few drug and food interactions and the dosage is fixed, offering important benefits that could impact patients’ health-related QoL (HRQoL) [18]. However, studies of HRQoL in patients with AF taking oral anticoagulants (NOACs vs. VKAs) are limited and the results have been heterogeneous [19–22]. It is also important to note that poorly controlled anticoagulation is very common (even more prevalent in real-life practice [23] than in controlled trials [24]) and might influence HRQoL [25]. However, the majority of the studies on AF patients have focused on assessing HRQoL according to treatment type (VKAs vs. NOACs), and there is a paucity of research determining the impact of INR control, and not treatment per se, on HRQoL. Characterisation of HRQoL according to anticoagulation control is important when tailoring therapies for patients with non-valvular AF (NVAF) and may influence treatment strategies and compliance. The present study has been designed to describe HRQoL in patients with NVAF who received conventional VKAs with poorly controlled anticoagulation and those with controlled anticoagulation who received VKAs or NOACs in a real-world setting. Additionally, we sought to identify factors associated with the demographic and clinical profile of NVAF patients treated with VKAs with poorly controlled anticoagulation.

## Methods

This was an observational, multicentre and cross-sectional study, in which 47 internal medicine specialists at 38 Spanish hospitals participated. The study comprised a single visit to the Internal Medicine department, which coincided with one of the patient’s routine follow-up visits. During the study visit, patients were invited to participate in the study, gave their informed consent, and self-completed the HRQoL questionnaire. There was no study-specific diagnostic or therapeutic intervention. The inclusion period was 10 months, from April 2017 to January 2018. To avoid selection bias, patients were consecutively enrolled by investigators when they met all the inclusion and none of the exclusion criteria. Patients were included in a 2:1 ratio (2 patients with controlled anticoagulation per 1 patient with uncontrolled anticoagulation), as patients with controlled anticoagulation comprise two treatments. The study was approved by the Ethics Committee of the Hospital Costa del Sol (Malaga, Spain) and by 21 additional Ethics Committees, and reported to the Spanish Health Authorities. The study was performed in accordance with the ethical standards of the 1964 Declaration of Helsinki and its subsequent amendments or comparable ethical standards.

The inclusion criteria were: patient diagnosed with NVAF, aged ≥ 18 years, on the same anticoagulant therapy (VKAs or NOACs) for at least 6 months and a maximum of 2 years, with the time in therapeutic range (TTR) available in previous analytical records or enough INR measures to calculate it (if treated with VKAs), and who provided informed consent to participate in this study. The exclusion criteria were: simultaneous participation in any clinical trial with a medicinal product or medical device and contraindication to the use of NOACs or VKAs as described in the summary of product characteristics.

Patients were divided into two groups as controlled or uncontrolled according to their anticoagulation control status. Patients treated with VKAs were classified as controlled if the TTR was ≥ 65% by the Rosendaal method [26] or by the direct method [27] when TTR was ≥ 60%. If % TTR was not available in analytical records, the internal medicine specialist calculated it using INR values from the previous 6 months (a minimum of four INR values were required). To perform the calculation, avoiding periods of anticoagulation initiation and treatment interruptions due to surgery or bleeding episodes was recommended. Patients treated with NOACs were classified as controlled, assuming that, as there is no need of TTR monitoring (in contrast to VKA treated patients) they were receiving the appropriate dose and, therefore, their anticoagulation status was controlled. As the present study was non-interventional and it only reflected interventions conducted per routine clinical practice, NOAC monitoring was not available.

HRQoL was assessed using the validated Spanish version [28] of the Sawicki questionnaire [29]. This questionnaire was designed to assess HRQoL in patients treated with oral anticoagulants and has been used in several studies [21, 30, 31]. It includes 32 items grouped in the following five dimensions: general treatment satisfaction, self-efficacy, strained social network, daily hassles and distress. Patients estimated the impact of anticoagulation treatment on each item of the questionnaire on a scale from 1 (total disagreement) to 6 (total agreement). The response options for each question were: 1 = not at all, 2 = very little, 3 = a little, 4 = somewhat, 5 = a lot, 6 = very much.

High self-perceived HRQoL is indicated by high scores in the general treatment satisfaction and self-efficacy dimensions, and inversely by low scores in the strained social network, daily hassles and distress dimensions. The summary score for each dimension was calculated by dividing the total score of the sum of the items of each dimension into the number of items included in that dimension.

Other data obtained during the study visit were: demographics (age, sex, race, work status, and marital status), physical exploration (height, weight and body mass index), NVFA history (time since diagnosis, age at diagnosis, time since treatment initiation, and type of NVFA), clinical data (creatine clearance, left ventricular ejection fraction [LVEF], CHA_2_DS_2_VASc score (6), HAS-BLED score (6), concomitant treatments and diseases, number of visits to the specialist, and history of thromboembolic and bleeding events) and treatment-related data (type of treatment, and TTR for patients treated with VKA). Creatinine clearance for assessment of kidney function was calculated using the Cockcroft–Gault formula.

Responses to the Sawicki questionnaire were coded and transformed into scales following the authors’ instructions. The main variable was the scores obtained in the five dimensions of the questionnaire by NVAF patients (quantitative variable), and this was expressed with measures of central tendency and measures of dispersion, including the mean and standard deviation (SD). The scores obtained for each of the 32 items were also described with the mean and SD. The secondary variables consisted in the demographic and clinical characteristics of patients with poorly controlled anticoagulation (uncontrolled patients). When these variables were quantitative, the mean and SD were presented. When the variables were qualitative, they were described by absolute and relative frequencies. In the descriptive analysis of the qualitative variables, two percentages were calculated: the total percentage (%) which was the percentage of the sum of valid responses plus missing values, and the valid percentage (% valid) which was the percentage of the total valid responses. The valid percentages (% valid) of the qualitative variables have been reported here. The statistical analyses were performed using SPSS v22.0.

## Results

**Fig. 1:**
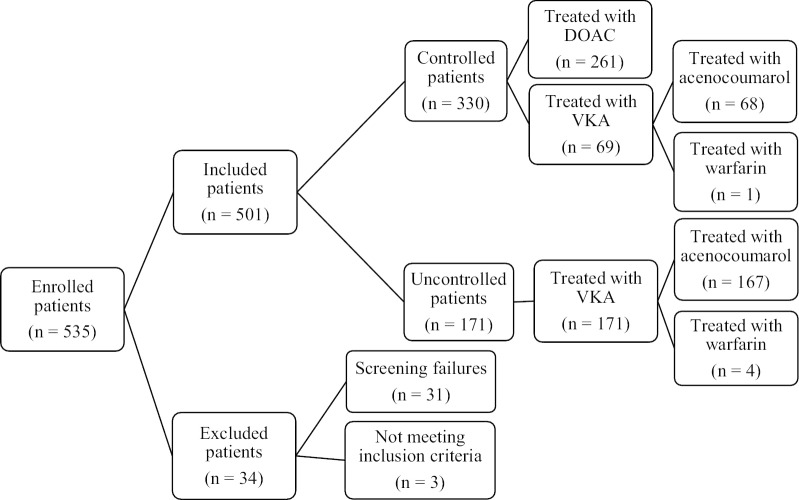
Disposition of study patients

Five hundred and thirty-five patients were enrolled. Thirty-four patients were excluded from the analysis (31 were considered screening failures because the expected sample size had been reached at the time of their inclusion, and 3 due to not meeting the inclusion criteria). The total number of patients included in the study was 501 (Fig. 1). All patients completed the HRQoL questionnaire. According to anticoagulation status, 330 patients were controlled (261 treated with NOACs and 69 treated with VKAs), and 171 patients were uncontrolled. In patients treated with VKAs, the mean ± SD % TTR was 90.3 ± 13.5% in controlled patients and 49.1 ± 10.8% in uncontrolled patients using the Rosendaal method, and 74.9 ± 10.4% in controlled patients and 35.0 ± 15.2% in uncontrolled patients using the direct method. The time (mean ± SD) in stable anticoagulation treatment was 14.0 ± 5.8 months in controlled patients and 14.8 ± 6.3 in uncontrolled patients.

Mean ± SD age was 79.7 ± 8.7 years, and 49.3% were women. Most of the patients were retired (73.3%) and 52.9% were married. Table 1 shows the demographic and clinical characteristics of patients treated with VKAs and NOACs according to anticoagulation control group.

**Table 1:** Demographic and clinical characteristics of NVAF patients according to anticoagulation status

Characteristics	Controlled patients			Uncontrolled patients	Total (*N* = 501)
	NOACs (*N* = 261)	VKA (*N* = 69)	Total (*N* = 330)	VKA (*N* = 171)	
Age (years, mean ± SD)	79.4 (8.6)	78.7 (9.4)	79.3 (8.8)	80.4 (8.7)	79.7 (8.7)
Gender					
Female (n, %)	119 (45.6)	33 (47.8)	152 (46.1)	95 (55.6)	247 (49.3)
Race (*n*, %)					
Caucasian	261 (100.0)	69 (100.0)	330 (100.0)	171 (100.0)	501 (100.0)
Age at diagnosis (years, mean ± SD)	74.9 (9.1)	75.8 (9.6)	75.0 (9.2)	77.3 (8.7)	75.8 (9.1)
Type of AF (n, %)					
Paroxysmal	84 (32.2)	18 (26.1)	102 (30.9)	51 (29.8)	153 (30.5)
Persisting	22 (8.4)	11 (15.9)	33 (10.0)	23 (13.5)	56 (11.2)
Permanent	155 (59.4)	40 (58.0)	195 (59.1)	56.1 (291)	291 (58.1)

*SD* standard deviation

Analysis of the data regarding the specific NVAF profile indicated that the mean ± SD time since diagnosis was 2.5 ± 3.2 years in uncontrolled patients and 4.0 ± 5.9 years in controlled patients, while the mean ± SD age at diagnosis was 77.3 ± 8.7 and 75.0 ± 9.2 years in uncontrolled and controlled patients, respectively. Time since initiating treatment was 14.0 ± 5.8 months in controlled patients and 14.8 ± 6.3 months in uncontrolled patients. The most common type of NVAF among patients was permanent (56.1% uncontrolled; 59.1% controlled), followed by paroxysmal (29.8% uncontrolled; 30.9% controlled), and persisting (13.5% uncontrolled; 10% controlled). The most common type of NVAF in both groups according to age was permanent in patients > 80 years (65.3% controlled; 62.9% uncontrolled), in patients between 75 and 80 years (62.9% controlled; 58.3% uncontrolled) and in patients between 65 and 74 years (47.1% controlled; 43.8% uncontrolled), and it was paroxysmal in patients between 18 and 64 years (50% controlled; 70% uncontrolled).

**Fig. 2:**
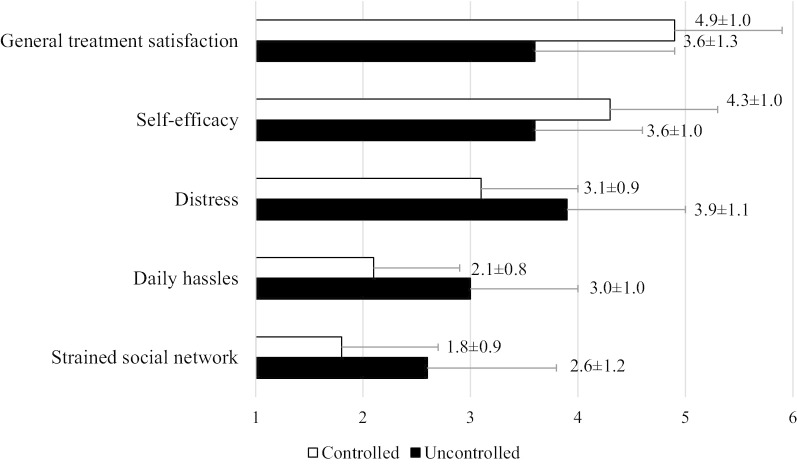
Mean ± SD scores in the dimensions of the Sawicki questionnaire for controlled and uncontrolled NVAF patients

Figure 2 shows mean ± SD scores in the five dimensions of the Sawicki questionnaire for controlled and uncontrolled patients. Overall, mean scores were closer to a high HRQoL in controlled patients than in uncontrolled patients in every dimension of the questionnaire. Mean scores for all individual items of the questionnaire are shown in Table 2. Post hoc analysis of the controlled patients treated with NOAC (n = 261) revealed similar mean ± SD) scores to all controlled patients (under NOACs and VKAs) in all the five dimensions: 5 ± 0.9 for general treatment satisfaction, 4.3 ± 1.1 for self-efficacy, 2 ± 0.8 for distress, 2 ± 0.8 for daily hassles and 1.7 ± 0.8 for strained social network.

**Table 2:** Mean ± SD scores in each item of the Sawicki questionnaire (grouped by dimensions) for controlled and uncontrolled NVAF patients

Items grouped by dimensions	Controlled patients (*N* = 330)	Uncontrolled patients (*N* = 171)
General treatment satisfaction^a^		
I am dissatisfied with the amount of time I invest in controlling my clotting	5.1 (1.4)	3.5 (1.7)
I am dissatisfied with the time it takes to get results	5.0 (1.4)	3.4 (1.6)
I dislike having to plan my activities in advance	4.8 (1.3)	3.6 (1.6)
I am worried by the uncertainty I feel while awaiting results	4.8 (1.6)	3.6 (1.7)
I am fed up with the amount of time I lose at the doctor’s surgery	4.5 (1.5)	3.3 (1.7)
I am annoyed that many people do not understand the problems related to my treatment	4.9 (1.4)	4.1 (1.6)
Self-efficacy		
I believe I have learned to control my treatment	4.3 (1.4)	3.6 (1.5)
I can deal with the treatment-related problems that arise	4.2 (1.3)	3.5 (1.3)
I am well informed about what to do to achieve results within the acceptable limits	4.4 (1.3)	4.0 (1.2)
I am sure I am able to control my treatment	4.3 (1.5)	3.4 (1.4)
Distress		
My treatment makes me feel worried or stressed	2.2 (1.4)	3.5 (1.7)
My treatment is a cause of concern for my family	3.0 (1.6)	4.2 (1.5)
I am worried about my future health	4.4 (1.5)	4.7 (1.3)
I am worried that my treatment may shorten my life	2.9 (1.7)	3.8 (1.8)
I feel dependent on my anticoagulation medication	3.2 (1.7)	4.2 (1.5)
I tend to worry about things	4.2 (1.4)	4.4 (1.3)
Despite regular visits to the doctor I feel limited	2.9 (1.5)	3.6 (1.5)
When I go to the dentist or other doctors, I am concerned that they might not know enough about anticoagulation	3.2 (1.5)	3.8 (1.6)
I dislike being treated like an invalid	2.5 (1.6)	2.9 (1.6)
I am worried about the side effects of my anticoagulant treatment	2.9 (1.5)	3.9 (1.6)
Daily hassles		
The effort of controlling my blood clotting causes me discomfort when I leave the house	1.8 (1.2)	3.6 (1.7)
I avoid some activities (e.g. cycling) due to the risk of accidents	2.5 (1.6)	3.3 (1.6)
My treatment prevents me from organizing my leisure time as I wish	1.7 (1.2)	3.2 (1.7)
The risk of cutting myself prevents me from doing housework	2.5 (1.5)	3.5 (1.5)
I am afraid of doing exercise out of fear to hurt myself	2.7 (1.6)	3.6 (1.6)
I would do more sports if I did not take anticoagulants	2.1 (1.3)	2.3 (1.3)
I have problems at work because of frequent absences caused by my treatment	1.2 (0.7)	1.5 (1.1)
Strained social network		
I see my friends less often since following this treatment	1.8 (1.2)	2.5 (1.6)
I avoid going on holiday because I do not know the negative effects different foods may have on treatment	1.9 (1.3)	2.8 (1.6)
I avoid travelling because I fear that I may not receive suitable treatment in case my results are too low or high	2.1 (1.4)	3.2 (1.6)
The treatment has affected my sex life	1.5 (1.0)	1.9 (1.3)
I am worried about other people´s reactions to my treatment	1.8 (1.2)	2.5 (1.4)

*SD* standard deviation

^a^Scores of items in the general treatment satisfaction dimension have been inverted

The clinical profile of uncontrolled patients is shown in Table 3. Data for all selected variables were not always available for each patient (n = 171), and therefore, the number of patients included in the analysis has been specified for each variable in Table 3. Briefly, mean ± SD values were 57.2 ± 26.6 ml/min for creatine clearance, 4.5 ± 1.4 points for the CHA_2_DS_2_-VASc index, and 3.6 ± 1.1 points for the HAS-BLED score. 60 (35.1%) uncontrolled patients had previously suffered a thromboembolic event, and 25 (14.6%) had a history of haemorrhagic events. The percentage of uncontrolled patients with at least one other disease recorded in the medical history was 98.8% and hypertension was the most common (85.8%) among those with comorbidities. Most uncontrolled patients (97.1%) were receiving a concomitant treatment, with furosemide being the most common (39.2%). The mean ± SD number of visits to the internal medicine specialist was 3.1 ± 1.9 visits per year.

**Table 3:** Characteristics of uncontrolled NVAF patients (treated with VKAs)

	Uncontrolled patients	Patients with data (*N*)
BMI (kg/m^2^, mean ± SD)	28.7 (5.4)	142
Creatine clearance (ml/min, mean ± SD)	57.2 (26.6)	150
> 80 years	50.9 (21.5)	
75–80 years	56.4 (14.4)	
65–74 years	73.5 (36.5)	
18–64 years	74.3 (37.6)	
Quantitative LVEF (%, mean ± SD)	56.1 (11.2)	108
Qualitative LVEF [n (%)]		161
Normal (≥ 50%)	140 (87)	
Slightly depressed (49–41%)	5 (3.1)	
Moderately depressed (40–31%)	10 (6.2)	
Severely depressed (≤ 30%)	6 (3.7)	
CHA_2_DS_2_-VASc score (mean ± SD)	4.5 (1.4)^a^	171
> 80 years	4.8 (1.2)	
75–80 years	4.7 (1.4)	
65–74 years	3.7 (1.2)	
18–64 years	3.1 (1.2)	
HAS-BLED score [n (%)]		171
Low-medium risk (score < 3)	27 (15.8)	
> 80 years	12 (11.4)	
75–80 years	3 (12.5)	
65–74 years	5 (15.6)	
18–64 years	7 (70.0)	
High risk (score ≥ 3)	144 (84.2)	
> 80 years	93 (88.6)	
75–80 years	21 (87.5)	
65–74 years	27 (84.4)	
18–64 years	3 (30.0)	
History of thromboembolic event [n (%)]	60 (35.1)	171
History of haemorrhagic event [n (%)]	25 (14.6)	171
Comorbidities [n (%)]	169 (98.8)	171
Hypertension	145 (85.8)	
Congestive heart failure	82 (48.5)	
Diabetes mellitus	65 (38.5)	
Renal failure	58 (34.3)	
Anaemia	55 (32.5)	
Arterial vascular disease	33 (19.5)	
Previous stroke/transient ischaemic attack	30 (17.8)	
Other^b^	67 (39.6)	
Concomitant treatments [n (%)]^c^	166 (97.1)	171
Furosemide	67 (39.2)	
Bisoprolol	46 (26.9)	
Atorvastatin	34 (19.9)	
Omeprazole	34 (19.9)	
Enalapril	30 (17.5)	
Metformin	24 (14.0)	
Allopurinol	20 (11.7)	
Digoxin	18 (10.5)	
Number of visits to the internal medicine specialist per year (mean ± SD)	3.1 (1.9)	171

*BMI* body mass index, *LVEF* left ventricular ejection fraction, *SD* standard deviation

^a^All patients (*n* = 171) had medium–high risk (scores ≥ 2)

^b^Other comorbidities affecting less than 5% of the patients

^c^Only concomitant treatments prescribed to more than 10% of the patients are reported

## Discussion

The present study provides valuable findings on HRQoL of NVAF patients under oral anticoagulant treatment with controlled and uncontrolled anticoagulant status in routine clinical practice. Scores in the five dimensions of the Sawicki questionnaire showed a better HRQoL in controlled than uncontrolled patients (controlled patients had higher scores in the satisfaction and self-efficacy dimensions, and lower scores in the distress, daily hassles and strained social network dimensions). The descriptive analysis revealed that controlled patients had ‘a lot’ of general treatment satisfaction, perceiving themselves to have ‘somewhat’ to ‘a lot’ of self-efficacy. Meanwhile, as regards general treatment satisfaction, uncontrolled patients were ‘a little’ to ‘somewhat’ satisfied. Scores were also ‘a little’ to ‘somewhat’ for uncontrolled patients’ self-efficacy perception. The impact of anticoagulant treatment on the distress dimension was ‘little’ in controlled patients and ‘somewhat’ in uncontrolled patients. Strained social network and daily hassles dimensions were ‘very little’ affected in controlled patients and ‘a little’ affected in uncontrolled patients. Therefore, controlled patients not only obtained scores indicating a better HRQoL for the general treatment satisfaction and self-efficacy dimensions, but also for the other dimensions (distress, daily hassles and strained social network).

Lower perceived HRQoL in patients treated with VKAs compared to those treated with NOACs has been previously reported [14, 21, 32, 33]. However, the initial differences in HRQoL observed between the two groups disappeared after 6 [21] and 12 months [25], suggesting a progressive adaptation to treatment with VKAs. Our study was cross-sectional and therefore did not assess whether HRQoL progresses differently in uncontrolled and controlled patients. However, the occurrence of HRQoL changes in our patients seems unlikely, since both the controlled and uncontrolled patients had been on anticoagulant treatment for more than 1 year on average, which can be considered enough time to adapt to the specific treatment.

The lack of differences between patients’ HRQoL could be also due to whether the questionnaire specifically comprised dimensions pertaining to anticoagulation or if it was a generic health status instrument to assess overall QoL. In fact, the selection of an appropriate HRQoL questionnaire has proven to be crucial. Both increases and decreases in HRQoL have been observed in the same patients depending on whether a generic or anticoagulation-specific questionnaire was used, respectively [17]. In line with this, greater differences have been observed between patients treated with VKAs or NOACs when HRQoL was assessed by a specific questionnaire such as the anti-clot treatment scale (ACTS) [12, 29] or the SAFUCA questionnaire [32] rather than a generic instrument like the EQ-5D [19, 25]. The lack of group differences found in some studies, probably due to the low degree of sensitivity of generic questionnaires, reinforces the need for specific tools to assess HRQoL in AF patients.

To the best of our knowledge, this is the first real-world data study that describes the HRQoL of NVAF patients undergoing anticoagulant treatment according to their anticoagulation control status and not their anticoagulant treatment per se*.* It is important to note that the patients had been on a stable anticoagulant regimen for more than 1 year, which constitutes one of the strengths of our study.

Poor anticoagulation control status (TTR < 50%) has been associated with the AF patient’s perception of fewer benefits of anticoagulation and greater emotional distress, specific concerns and burdens of therapy [34]. Our study confirmed that patients with uncontrolled anticoagulation (TTR < 65%) reported lower HRQoL compared to patients with controlled anticoagulation. These results were expected given the known complexities of VKA treatment. Experiencing out-of-range INR results, dose changes, diet restrictions and more frequent visits to the physician might have given rise to the patient’s perception of their illness as a burden.

Our study also described the demographic and clinical characteristics of NVAF patients treated with VKAs with uncontrolled TTR in Spain. Among these uncontrolled patients, the percentage of women was slightly higher (55.6%) than men, a pattern that was not observed in our controlled patients. Accordingly, being female has been shown to increase the risk of AF in general [9] and to be associated with a higher risk of poor INR control in particular [15, 35, 36]. Female gender has been also independently associated with reduced QoL and increased AF symptoms [37]. Programmes aimed at improving oral anticoagulation control should also consider the role played by gender [15].

The percentage of patients over 80 years of age was higher in the uncontrolled than controlled group (61.4 vs. 56.1). Older age has been shown to influence anticoagulation control, with TTR declining after 67 years of age [38]. The higher percentage of older patients in the uncontrolled group might have contributed to lower HRQoL scores in this group, due to the presence of multiple comorbidities including dementia, a tendency to falls and frailty, chronic kidney disease, hypertension, and diabetes. Thromboembolic risk in our uncontrolled patients was comparable to that observed in studies where the mean age of AF patients was between 77 and 78 years [14, 15], and higher when compared to studies with younger patients (mean age 63–74 years) [13, 19, 21]. Bleeding risk, on the other hand, was higher in our patients than in other studies with AF patients, irrespective of the mean age of the sample [13–15, 19, 21]. The thromboembolic and bleeding risks found in our uncontrolled sample were higher than in other studies with uncontrolled AF patients with TTR < 50% [34]. It should be emphasized here that higher thromboembolic and bleeding risks might have been due to not only the lack of anticoagulation control, but also because of the presence of modifiable bleeding risk factors such as hypertension, concomitant antiplatelet therapy, nonsteroidal anti-inflammatory drugs use and alcohol abuse.

As indicated in previous studies, the majority of AF patients have hypertension as their most common comorbidity [6, 11, 14, 21, 39]. The strong link between AF and hypertension has been also underlined in a study conducted in Spain where 10.3% of patients with hypertension who were older than 65 years had AF [40].

The results of our study should be considered taking into account some limitations. First of all, patients treated with NOACS were included in the “controlled patients” group, assuming that they received an appropriate dose and were compliant with the treatment in contrast to VKA treated patients, which need TTR monitoring to determine if they are controlled or not. Even though the work of Hwang et al. [41]. determine that NOAC treated patients compliance is excellent, other studies suggest the adherence is suboptimal [42, 43], and therefore this assumption should be addressed as a study design limitation. Secondly, the questionnaire used was originally designed for assessment of HRQoL in patients treated with VKAs. In spite of the fact that some aspects assessed by the Sawicki questionnaire are specific to VKA treatment (i.e. items related to treatment control) this limitation is unlikely to have had an impact on our results, since it has been used in previous studies reporting comparable HRQoL scores [21]. Additionally, the analyses between groups using statistical tests were not performed, and conclusions regarding differences between groups in HRQoL should be regarded with caution. Nevertheless, the study allows us to describe the HRQoL perceptions of the studied population, which was the primary objective of the study. Finally, there are some factors other than anticoagulation control that could influence QoL in AF patients, such as symptoms or correlated comorbidities, which were not taken into account on this study and could be of interest for further research.

## Conclusions

In conclusion, the present study shows that patients with controlled anticoagulation status (treated with either NOACs or VKAs) had better mean scores for perceived HRQoL in all the dimensions of the Sawicki questionnaire than patients with uncontrolled anticoagulation status. Moreover, we have provided a detailed description of the demographic and clinical profile of uncontrolled NVAF patients on anticoagulants, identifying particular characteristics that might influence HRQoL perception and which should be evaluated. An understanding of the factors that influence HRQoL is required in order to adapt economic evaluations to the individual and specific characteristics of every NVAF patient, assessing the clinical and cost-effectiveness of the different therapeutic alternatives.

## Data Availability

Please contact author for data requests.

## Abbreviations

AF: Atrial fibrillation
HRQoL: Health-related quality of life
INR: International normalized ratio
LVEF: Left ventricular ejection fraction
NVAF: Nonvalvular atrial fibrillation
NOACs: Non-vitamin K antagonists oral anticoagulant
QoL: Quality of life
TTR: Therapeutic time in range
VKAs: Vitamin K antagonists
