# Correction to: Health-related quality of life in nonvalvular atrial fibrillation patients with controlled or uncontrolled anticoagulation status

**DOI:** 10.1186/s12955-021-01751-7

**Published:** 2021-04-01

**Authors:** José Felipe Varona, José Miguel Seguí‑Ripoll, Cristina Lozano‑Duran, Luis Miguel Cuadrado‑Gómez, Juan Bautista Montagud‑Moncho, Antonio Ramos‑Guerrero, José Carlos Mirete‑Ferrer, Esther Donado, Javier García‑Alegría, the REQUOL Study Group

**Affiliations:** 1 Departamento de Medicina Interna, Hospital Universitario HM Montepríncipe, HM Hospitales, Madrid, Spain; 2 Hospital San Juan de Alicante, San Juan de Alicante, Alicante Spain; 3 Department of Clinical Medicine, Miguel Hernández University, Elche, Alicante Spain; 4 Hospital Universitario Príncipe de Asturias, Alcalá de Henares, Madrid Spain; 5 Hospital Francesc de Borja, Gandía, Valencia Spain; 6 Hospital San Juan de Dios del Aljarafe, Bormujos, Seville Spain; 7 Hospital de Torrevieja, Torrevieja, Alicante Spain; 8 Boehringer-Ingelheim, Sant Cugat del Vallés, Barcelona Spain; 9 Hospital Costa del Sol, A‑7, Km 187, 29603 Marbella, Malaga Spain

## Correction to: Health Qual Life Outcomes (2020) 18:383 10.1186/s12955-020-01563-1

The original article contains an error in the description of co-author, José Felipe Varona’s affiliation.

The correct affiliation can be viewed in this Correction article.
